# HERC5 downregulation in non-small cell lung cancer is associated with altered energy metabolism and metastasis

**DOI:** 10.1186/s13046-024-03020-z

**Published:** 2024-04-11

**Authors:** Svenja Schneegans, Jana Löptien, Angelika Mojzisch, Desirée Loreth, Oliver Kretz, Christoph Raschdorf, Annkathrin Hanssen, Antonia Gocke, Bente Siebels, Karthikeyan Gunasekaran, Yi Ding, Leticia Oliveira-Ferrer, Laura Brylka, Thorsten Schinke, Hartmut Schlüter, Ilkka Paatero, Hannah Voß, Stefan Werner, Klaus Pantel, Harriet Wikman

**Affiliations:** 1 Department of Tumor Biology, University Medical Center Hamburg-Eppendorf, Martinistraße 52, 20246 Hamburg, Germany; 2 III. Department of Medicine, University Medical Center Hamburg-Eppendorf, Hamburg, Germany; 3 Section Mass Spectrometry and Proteomics, University Medical Center Hamburg-Eppendorf, Hamburg, Germany; 4 Center for Molecular Neurobiology (ZMNH), University Medical Center Hamburg- Eppendorf, Hamburg, Germany; 5 Department of Biochemistry and Molecular Cell Biology, University Medical Center Hamburg-Eppendorf, Hamburg, Germany; 6 Department of Gynecology, University Medical Center Hamburg-Eppendorf, Hamburg, Germany; 7 Department of Osteology and Biomechanics, University Medical Center Hamburg-Eppendorf, Hamburg, Germany; 8 Turku Bioscience Centre, University of Turku and Åbo Akademi University, Turku, Finland

**Keywords:** HERC5, NSCLC, Metastasis, DTC, Cancer metabolism, OXPHOS, Mitochondria, Metastasis, Warburg effect

## Abstract

**Background:**

Metastasis is the leading cause of cancer-related death in non-small cell lung cancer (NSCLC) patients. We previously showed that low HERC5 expression predicts early tumor dissemination and a dismal prognosis in NSCLC patients. Here, we performed functional studies to unravel the mechanism underlying the “metastasis-suppressor” effect of HERC5, with a focus on mitochondrial metabolism pathways.

**Methods:**

We assessed cell proliferation, colony formation potential, anchorage-independent growth, migration, and wound healing in NSCLC cell line models with HERC5 overexpression (OE) or knockout (KO). To study early tumor cell dissemination, we used these cell line models in zebrafish experiments and performed intracardial injections in nude mice. Mass spectrometry (MS) was used to analyze protein changes in whole-cell extracts. Furthermore, electron microscopy (EM) imaging, cellular respiration, glycolytic activity, and lactate production were used to investigate the relationships with mitochondrial energy metabolism pathways.

**Results:**

Using different in vitro NSCLC cell line models, we showed that NSCLC cells with low *HERC5* expression had increased malignant and invasive properties. Furthermore, two different in vivo models in zebrafish and a xenograft mouse model showed increased dissemination and metastasis formation (in particular in the brain). Functional enrichment clustering of MS data revealed an increase in mitochondrial proteins in vitro when HERC5 levels were high. Loss of *HERC5* leads to an increased Warburg effect, leading to improved adaptation and survival under prolonged inhibition of oxidative phosphorylation.

**Conclusions:**

Taken together, these results indicate that low HERC5 expression increases the metastatic potential of NSCLC in vitro and in vivo. Furthermore, HERC5-induced proteomic changes influence mitochondrial pathways, ultimately leading to alterations in energy metabolism and demonstrating its role as a new potential metastasis suppressor gene.

**Supplementary Information:**

The online version contains supplementary material available at 10.1186/s13046-024-03020-z.

## Background

Lung cancer is the most common cancer among men and the third most common cancer among women, causing approximately 1.8 million deaths worldwide, with an estimated 31.5% of new cases annually diagnosed in men and 14.6% in women in 2020 (age-standardized incidence rate) [1]. Despite the great benefits of targeted therapies and immune-therapies for some patients, the 5-year survival rate still ranges between 10 and 20% [1]. Thus, the identification of novel markers and targets involved in disease progression is an essential step in improving the survival of lung cancer patients. Previously, we reported that loss of HERC5 (HECT and RLD domain containing E3 ubiquitin protein ligase 5) in non-small cell lung cancer (NSCLC) patients leads to a positive disseminated tumor cell (DTC) status in the bone marrow of patients with early-stage NSCLC as well as brain metastasis formation and poor overall survival (OS) [2], indicating a potential metastasis-suppressing function of HERC5. Additionally, in hepatocellular and colorectal cancer, low HERC5 expression was correlated with shorter tumor recurrence and OS [3, 4]. On the molecular level, these studies showed that HERC5 loss could drive immunosuppression and tumor progression. Furthermore, in several different tumor entities, HERC5 mRNA expression, often as part of a signature, has been correlated with diagnostic and prognostic characteristics in different tumor entities [3–8], indicating a potentially important role of HERC5 in metastasis.

Originally described in the context of innate immune responses, HERC5 is known to serve as the main cellular E3 ligase for ubiquitin-like modifier interferon-stimulated gene 15 (ISG15) [9, 10], thereby catalyzing the transfer of ISG15 to target proteins—a process called ISGylation. In addition to being an important primary defense mechanism against viruses and other pathogens [11, 12], ISGylation has been implicated in several diseases, such as immunodeficiency, neurodegenerative disorders, and different types of cancer [13–16]. Deregulation of ISG15 has been linked to dysfunctional mitochondria as well as altered OXPHOS activity [17–20]. Energy metabolism strongly influences tumorigenesis and metastasis formation [21–25]. Given the enhanced energy demand of highly proliferating cells, tumor cells can change energy consumption mechanisms, such as the well-described Warburg effect, leading to a switch to ATP production through glycolysis rather than oxidative phosphorylation (OXPHOS) [26]. Despite the decreased yield of ATP through glycolysis, malignant cells often prefer this pathway even in the presence of oxygen, leading to an increase in metabolic byproducts, which are necessary for the elevated demand for metabolites due to their high proliferation rate [21, 22].

In this study, we aimed to further elucidate the potential metastasis suppressing role of HERC5 in NSCLC. Therefore, we assessed the malignant potential of HERC5 loss in in vitro and in vivo NSCLC models and demonstrated its capacity to transform NSCLC cells both in vitro and in vivo into a more aggressive phenotype. Furthermore, we showed that HERC5 influences mitochondrial protein composition, morphology, and energy metabolism pathways, such as OXPHOS, leading to improved adaptation and survival under prolonged inhibition of oxidative phosphorylation.

## Methods

### Cell culture

All cell lines used in this study were obtained from the ATCC. Authentication was performed by the Multiplex Human Cell Authentication (MCA) test to exclude cross-contamination between the cell lines, which was last confirmed on July 18th, 2022. All cells were grown as monolayers to a confluence of approximately 90% before subculturing. A549 cells were grown in DMEM (PAN Biotech, DE) supplemented with 10% FCS (PAN Biotech, DE), 2 mM L-glutamine (PAA Laboratories, AT), and 200 U/mL Pen/Strep (200 U/mL; Thermo Fisher Scientific, US) in 10% CO_2_ at 37 °C. HTB56 cells were grown in MEM (Gibco BRL, Thermo Fisher Scientific, US) supplemented with 10% FCS, 2 mM L-glutamine, 200 U/mL Pen/Strep, 1% NEAA (MEM nonessential amino acid solution; Sigma‒Aldrich, US), and 1% sodium pyruvate (Gibco BRL, Life Technologies, DE) and incubated in 5% CO_2_ at 37 °C.

### Generation of HERC5-overexpressing and -luciferase-expressing cell lines

HEK293T cells at approximately 80% confluency were transfected with 3 µg of either the LEGO-iG2-HERC5-HA or LEGO-iG2-HA plasmid for overexpression or 5 µg of the pHIV-luciferase plasmid (Addgene, US), 3750 ng of the psPAX2 packaging plasmid (Addgene, US) and 1250 ng of the pMD2.D envelope plasmid (Addgene, US), 20 µL of Lipofectamine 2000 (Invitrogen, US) reagent and 500 µL of OptiMEM (Gibco, US). The transfection mixture was replaced with standard medium 12–15 h after transfection. The virus-containing supernatant was collected after an additional 48 h and filtered using 0.45 µm syringe filters (Millex, DE). Viral stocks were stored at -80 °C. 200 µL of lentiviral particles were added dropwise to cultured HTB56 cells at approximately 80% confluency in media containing polybrene (1:1000, Fluka (Thermo Fisher Scientific, US)) to enhance transduction efficiency. 24 h after viral transduction, the media was changed. Cells overexpressing HERC5 or empty vector control were sorted by fluorescence-activated cell sorting (FACS) at 488 nm and recultured for further experiments.

### Generation of a HERC5 knockout cell line

A549 HERC5 KO cells were generated by using clustered regularly interspaced short palindromic repeat (CRISPR)/CRISPR-associated nuclease 9 (Cas9) technology. For efficient knockout of the HERC5 gene, different CRISPR guides were tested for efficiency and cloned and inserted into pSpCas9(BB)-2A-GFP (PX458, Addgene, US). Finally, sgRNA_HERC_5_sense (CACC GCGCAACGGGCGCTCGACCGC) and sgRNA_HERC_5_antisense (AAAC GCGGTCGAGCGCCCGTTGCGC) were chosen. After A549 cells were transfected with Lipofectamine 2000 according to the manufacturer’s protocol, successful HERC5 KO was verified by Western Blotting and Sanger sequencing of individual expanded cell clones. After 5 days, single GFP-positive cells were isolated using fluorescence-activated cell sorting (FACS) and clonally expanded. To minimize off-target effects, a pool of four different KO clones was selected to generate the A549-HERC5 KO cell line.

### Western blotting

To generate whole-protein lysates, cells were grown to a confluence of 90%, washed with PBS, and detached from the culture flask surface. The pellet was resuspended in 2% SDS sample buffer containing Complete Protease Inhibitor (Roche Applied Science AG, DE) and PhosSTOP (Roche Applied Science AG, DE) phosphatase inhibitors and homogenized by sonication. A Pierce BCA Protein Assay Kit (Thermo Fisher Scientific, US) was used according to the manufacturer’s instructions to quantify the protein concentration of the whole-cell extracts. SDS‒PAGE was performed using 8% and 4‒20% (NIPPON Genetics Europe, DE) polyacrylamide gels under denaturing conditions, after which the proteins were blotted on nitrocellulose membranes. Blocking was performed in 5% (w/v) milk powder/PBS-T or 5% (w/v) BSA/TBS-T for 1 h at RT. The following antibodies were used for detection: HERC5 (Abnova, TW; A01, 1:1000, o/n) and HSC70 (Santa Cruz Biotechnology, US; B-6, 1:2,000,000, o/n). For western blots depicting cellular responses to immune activation, the cell lines were treated with IFNγ (10 ng/ml; PeproTech, US) and TNFα (50 ng/ml; PeproTech, US) for 24 h and 48 h. Detection of the western blots was performed using ISG15 antibody (CST, US; #2743, 1:1000 o/n).

### Methylene blue proliferation assay

Commonly used proliferation assays (e.g., MTT assays) are dependent on the metabolic conversion of MTT by NAD(P)H oxidoreductases. Therefore, methylene blue assays were used to assess the proliferation rates of the cell lines according to Felice et al*.* (2009) [27]. For this purpose, either 1000 HTB56 or 500 A549 cells were seeded in 96-well plates and placed in an incubator until the day of the measurement at days 1 (A549), 2 (HTB56), 4, 6, and 8 (both cell lines). Cells were treated with 100 ng/mL oligomycin (A549) according to Hao et al*.* (2010) [28] and 1 ng/mL oligomycin (HTB56). For detection, the cell culture supernatant was discarded, and the cells were washed with 300 µL of PBS. 50 µL of staining solution (1.25% glutaraldehyde and 0.6% methylene blue in Hanks Balanced Salt Solution (Hanks Salt Solution [Biochrom GmbH, DE], 0.8 mM MgSO4, 1.3 mM CaCl_2_) was added to each well, and the mixture was incubated for 1 h at 37 °C. The staining solution was discarded, and each well was washed by rinsing the plate six times in dH_2_O. After drying, 100 µL of elution solution (50% ethanol and 1% acetic acid in PBS) was added to each well and was also used as a blank. Methylene blue was eluted from the cells by shaking on a plate shaker (450 RPM) for 15 min. The absorbance was read at 600 nm in a plate reader (GloMax Discover, Promega, US).

To evaluate differences in proliferation ratios, the absolute values on day 8 and days 1 (A549) and 2 (HTB56) were calculated, and the relative growth of the cell lines was assessed. The groups were compared by one sample t test.

### Clonogenic assay

To determine the ability of single cells to grow into a colony, 1000 cells were seeded in 6-well plates and imaged after 7 days. After removal of the media, the cells were washed with PBS, and the colonies were fixed with 4% (w/v) PFA/PBS for 10 min. Subsequently, the colonies were stained with 0.5% (w/v) crystal violet/H_2_O solution for 30 min and gently washed with ddH_2_O to remove excess staining solution. Colonies were detected using the ColonyArea plugin for ImageJ [29].

### Anchorage-independent growth assay

To produce the bottom agar solution, 2 mL of cell culture media and heated agar solution (42 °C; final concentration: 0.5% in ddH_2_O) were mixed, poured into 6-well plates, and left to harden. For each well, 1 mL of top agar was layered on top of the bottom agar, consisting of 2/3 bottom agar and a 1/3 cell suspension (3000 cells). After 14 days, 200 µL of MTT was added dropwise to the wells, and the plate was imaged after 3 h. Colonies exceeding a diameter of 100 µm were counted using the Cell Colony Edge macro for ImageJ [30]. The detection parameters were altered to “Number of pixels/units” = 0.0945; “Rolling Ball Radius” = 50; “Analyze Particles—Min size” = 7854 and “Analyze Particles—Min circ” = 0.5.

### Migration assay

The transwell migration assay was performed by plating 500 µL of FCS-deprived media containing 20,000 cells into cell culture inserts (BD Falcon, DE; 8 µm pores), while media containing 10% FCS as a chemoattractant was placed in the lower chambers. After 16 h, the media was removed, and the membrane was washed in PBS and fixed with 4% (w/v) PFA/PBS for 10 min. The cells were permeabilized with methanol for 20 min and stained with 0.5% (w/v) crystal violet/H_2_O solution for 10 min. The excess staining solution was removed by washing in PBS. Nonmigrated cells were scraped from the inside of the inserts with sterile cotton. The membrane was imaged under a brightfield microscope (Zeiss, Axiovision), and the cells were counted manually.

### Wound healing assay

For each cell line, 1*10^6^ cells were plated in 6-well plates and incubated at 37 °C until a confluent monolayer was formed. Using a 100 µL pipette tip, straight scratches were made in the middle of the well. The cells were washed with PBS to remove cell debris and covered in culture medium. The scratches were imaged at a defined position at 0 h and after 18 h of incubation to analyze the extent of wound healing that had taken place. The results were analyzed using the “Wound_healing_size_tool” plugin for ImageJ [31]. The mitochondrial activity was visualized using MitoTracker™ Green FM and MitoTracker™ Red CMXRos kits according to the manufacturers’ instructions (Thermo Fisher Scientific, US) using 20 nM for 30 min for both dyes.

### In vivo zebrafish experiments

Zebrafish experiments were carried out in the Zebrafish Core of Turku Bioscience Centre (Turku, Finland). The overall process of generating zebrafish xenografts has been described in detail earlier [32]. In brief, zebrafish embryos of the Casper strain were obtained through natural spawning. The collected eggs were cultured in E3 + PTU media. Before injection at 2 dpf, the embryos were dechorionated, anesthetized, and mounted in low-melting point agarose. Tumor cells were injected into the common cardinal vein at 2 days postfertilization (dpf). After injection, the embryos were released from the agarose and cultured in a 33 °C incubator in E3 + PTU supplemented with penicillin‒streptomycin. At 1 day postinjection (dpi), the embryos were anesthetized, placed in a 96-well plate (1 embryo/well), and imaged using a Nikon Eclipse Ti2 wide-field microscope. After imaging, the anesthetic was removed, and the embryos were cultured at 33 °C until imaging at 3 dpi. The number of extravasated (1 dpi) and survived (3 dpi) disseminated tumor cells (DTCs) was counted manually using QuPath (version 0.4.4). DTCs were counted separately for the brain, defined as the anatomical region situated anterior to both the pericardiac cavity and the otic vesicle, and for the body (all remaining anatomical structures). The samples were blinded during the image analysis.

### In vivo mouse experiments

Intracardial injection into the left ventricle of five 9-week-old nude mice (NMRI-Foxn1nu; Jackson Laboratories, US) per group was performed using 5*10^5^ A549-luciferase or A549-HERC5-KO-luciferase cells in 100 μL of 0.9% NaCl solution. Photon emission was measured after injection of 3 mg D-luciferin (Biosynth Carbosynth, UK) per mouse and incubation for 10 min (BLI). Mice were euthanized after 31 days. X-ray analysis of the whole mouse skeleton was performed, and blood and bone marrow samples were purified and stained by using immunofluorescence. Brain and adrenal gland tissues were formalin-fixed and paraffin-embedded (FFPE).

### Enrichment and detection of circulating tumor cells (CTCs) and DTCs from blood and bone marrow samples

Retrobulbar blood samples from each mouse cohort were pooled and processed with a size-based separation system (Parsortix™ Cell Separation System Angle plc, UK) for isolation of CTCs using 6.5 μm cassettes. Bone marrow was isolated from the femurs and tibias of the mice after mechanical cleaning of the muscles and other tissues and by opening on one side and placing the bones upside down in a reaction tube. After the addition of 10 μL of PBS, the bone marrow was flushed by centrifugation at 8000 × g for 30 s. After centrifugation at 16,000 × g for 10 min, the pellet was resuspended in 500 μL of 1 × Flow Cytometry Human Lyse Buffer (R&D Systems, US) and incubated for 3 min. PBS was added, and the mixture was centrifuged again at 16,000 × g for 10 min. The supernatant containing the PBMCs and DTCs was diluted in PBS and cytocentrifuged on SuperFrost® microscope slides (R. Langenbrinck GmbH, DE). CTCs and DTCs were detected by immunofluorescence staining using mouse CD45-APC (1:150; BD Pharmingen, US; 30-F11), pancytokeratin (1:200; Invitrogen, US; AE1/AE3-al570), and pankeratin (1:200; Cell Signaling, US; C11-al555) as described previously [33].

### Analysis of tumor spread in mice

The brain tissue was cut coronally into five slices, while the adrenal glands remained in one piece. The FFPE embedded organs were cut into 5 μm thin slices, and after deparaffinization, antigen retrieval products (BioGenex Citrate Buffer, pH 6; Thermo Fisher Scientific, US) and antibodies (Keratin AE3; EMD Millipore, DE; MAB1611 and keratin AE1; EMD Millipore, DE; MAB1612, both 1:1000) were diluted with Antibody Diluent DAKO incubated overnight in a humidified chamber at 4 °C and detected with a DAKO REAL Detection System (Agilent Technologies, US) according to the manufacturer’s protocol. The tumor volume of the brain and of the adrenal gland metastases were calculated by the following formula modified from Kunkel et al. [34]: $Tumor\;volume=\left[\sqrt[2]{\left(tumor\;surface\;area\right)}\right]^3$.

For analysis of brain metastases, every 10th slice was analyzed, corresponding to an interval of 50 μm.

### Mass spectrometry analysis

The cell pellets from untreated and treated (IFNγ (10 ng/ml; PeproTech, US) and TNFα (50 ng/ml; PeproTech, US) for 48 h) cells were resuspended in 500 μL of 0.1% TEAB/1% SDC for cell lysis and heated for 5 min at 95 °C. The samples were sonicated (6 pulses at 30%) using a sonication probe, and the protein concentration was analyzed (see Western Blotting). 20 μg of protein was tryptically digested in 100 μL of 0.1% TEAB/1% SDC, and disulfide bridges were reduced by 10 mM dithiotreithol for 30 min at 60 °C. After incubation with 20 mM iodacetamide for 30 min at 37 °C in the dark for alkylation of thiol groups, proteolytic cleavage after arginine and lysine residues was performed using a trypsin:protein ratio of 1:100 (37 °C overnight). SDC was removed by precipitation with 1% acetic acid, and the remaining supernatant was lyophilized using a SpeedVac vacuum concentrator and resuspended in 0.1% FA to a final concentration of 1 mg/mL. 1 μg of protein was injected into a Dionex Ultimate 3000 UPLC system. Peptides were purified and desalted using an Acclaim PepMap 100 C18 trap (100 μm × 2 cm, 100 Å pore size, 5 μm particle size) precolumn (Thermo Fisher Scientific, US) and transferred to an Acclaim PepMap 100 C18 analytical column (75 μm × 50 cm, 100 Å pore size, 2 μm particle size; Thermo Fisher Scientific, US) for chromatographic separation. The eluting peptides were subsequently transferred into a Quandrupole-Iontrap-Orbitrap tribrid mass spectrometer (Orbitrap Fusion, Thermo Fisher Scientific, US) and analyzed via a label-free DDA LC‒MS2 approach using an Orbitrap mass analyzer at the MS1 and an ion trap mass analyzer at the MS2 level. The mass spectrometry proteomics data have been deposited in the ProteomeXchange Consortium via the PRIDE [35] partner repository with the provisional dataset identifier PXD045563. The resulting mass spectra were searched against a reviewed human FASTA database (Swissprot) containing 20,365 entries downloaded in October 2020 using MaxQuant version 1.6.2. The carbamidomethylation of cysteine residues was set as a fixed modification. The oxidation of methionine was included as a variable modification. Matching between runs was included to increase the number of identified peptides in each sample. LFQ intensities were used for quantitative analysis via PERSEUS version 1.5.8.5., log2-transformed and median-normalized across columns.

Significantly differentially abundant proteins were identified using a two-sample Student’s t test. Proteins that had a *p* value < 0.05 and an additional fold change cutoff > 1.5 were considered to be significantly differentially abundant across groups.

### In Silico validation

For external validation of our findings publicly available proteome data of 141 lung cancer patients from Lehtiö et al. (2021) [36], accessible via PRIDE (PXD020548) was used. The preprocessed DDA data with a threshold of 0.5 and -0.5 was used to stratify patients in a HERC5 high (*n* = 24) and a HERC5 low (*n* = 14) group based on normalized HERC5 abundance exceeding a threshold of 0.5 (high) or -0.5 (low). As in the cell line data, significantly differential abundant proteins were identified using an unpaired Students T-Test. Proteins with a *p* – value < 0.05 and fold-change > 1.5 or <—1.5 were considered as significantly differential abundant across the two groups.

### GO term analysis and GO term enrichment analysis

Gene ontology plots were generated according to Bonnot et al. [37], here displaying the top 5 significant (FDR < 0.05) enriched GO terms. Using this list, the corresponding genes that make up the GO biological processes were visualized using the R package GOplot according to Walter et al. [38]. EnrichR [39] was used for the pathway enrichment analyses of the validation cohort using the GO Biological Process and GO Molecular Function databases and a ranking based on the calculated *p*-value.

### Electron microscopy imaging

For EM imaging, A546 PAR/KO and HTB56 EC/OE cell lines were cultivated in T75 flasks, trypsinized, and resuspended in 1 ml of 4% PFA plus 1% glutaraldehyde (Roth, DE) in 0.1 M PBS. The cells were fixed at RT for 1 h and subsequently at 4 °C overnight in the same fixative. After centrifugation, the resulting pellets were contrasted in 0.5% osmium tetroxide (30 min at RT), dehydrated, and embedded in epoxy resin (Durcupan, Sigma‒Aldrich, UK). Images were taken using a Philips CM100 transmission electron microscope. For quantification, random images of 22–31 cells per condition were taken and analyzed for mitochondrial density per cytoplasmatic area. Moreover, we measured mitochondrial size (338–581 mitochondria per condition) using the same set of random images. For the measurements, we used ImageJ software.

### NAD/NADH Glo-assay

The NAD/NADH-Glo™ Assay (Promega, US) was performed according to the manufacturer’s instructions. After the calculation of the NAD/NADH ratios, a two-sided Student’s t test was used to determine the significance of the differences between the cell lines.

### Seahorse XF assays

A549 PAR/KO or HTB56 EC/OE cells were plated in 96-well cell culture microplates (XFe96, Agilent Technologies, US) at a density of 10,000 cells per well. On the day of measurement, the cells were incubated for 1 h in bicarbonate-free DMEM containing 5 mM HEPES, 10 mM glucose, 1 mM pyruvate, and 2 mM glutamine. Oxygen consumption and extracellular acidification rates (OCR and ECAR) were measured simultaneously using a Seahorse XFe96 Flux Analyzer (Cell Mito Stress Test Kit and Glycolytic Rate Assay Kit, Agilent Technologies, US) according to the manufacturer’s instructions. ATP-dependent respiration was profiled by injecting 1.5 μM oligomycin (which inhibits ATP synthase), and the full substrate oxidation capacity was determined by injecting either 1.5 µM (A549 cells) or 0.75 µM (HTB56 cells) carbonylcyanide-p-trifluoromethoxyphenylhydrazone (FCCP, a chemical uncoupler). Nonmitochondrial respiration was determined by injecting 0.5 μM antimycin A and 0.5 μM rotenone (which inhibit electron flux through complexes I and III). Additionally, conducting the Glycolytic Rate Assay, basal glycolysis was measured followed by measuring the compensatory glycolysis by injecting 0.5 μM antimycin A and 0.5 μM rotenone. Finally, glycolysis was inhibited to confirm the experimental setup by injecting 50 mM 2-deoxy-glucose (2-DG, a glucose analog). OCRs and ECARs were determined by machine learning algorithms and plotted against time. The values were normalized to the DNA content by Quant-iT picoGreen staining (Thermo Fisher Scientific, US) for the Mito Stress Test and nuclei count determined by DAPI signal after fixation with 4% PFA and staining with 1 µg/mL DAPI using CellProfiler (version 4.2.6) [40] for Glycolytic Rate Assay. Relative ATP production rates were calculated from the OCR and ECAR rates assuming a P/O ratio of 2.75.

### Lactate assays

Lactate secretion by A549 cells during long-term oligomycin treatment was assessed with a Lactate-Glo™ Assay (Promega, US). The supernatants from eight biological replicates of the methylene blue proliferation assay were collected, and a lactate assay was performed according to the manufacturer’s instructions. The samples were diluted 1:500 in PBS. The reaction mixture was downscaled to 50 µL per reaction, and luminescence was measured in duplicate using a plate reader (GloMax Discover, Promega, US).

### Statistical analyses

All the experiments were performed using at least three independent biological replicates unless otherwise indicated. Statistical testing was performed using Student’s t test, one-sample t test, paired t test, Wilcoxon one-sample signed rank test, or Mann‒Whitney U test. The results were considered significant when the obtained p values were less than 0.05 (*). Significance values were obtained using Excel (Microsoft Office Professional 2013 Plus), R (version 4.0.3), R Studio (version 1.1.423), or GraphPad Prism (version 9.4.1).

## Results

### Enhanced tumorigenic properties are characteristic of HERC5 low-expressing cells in vitro

**Fig. 1:**
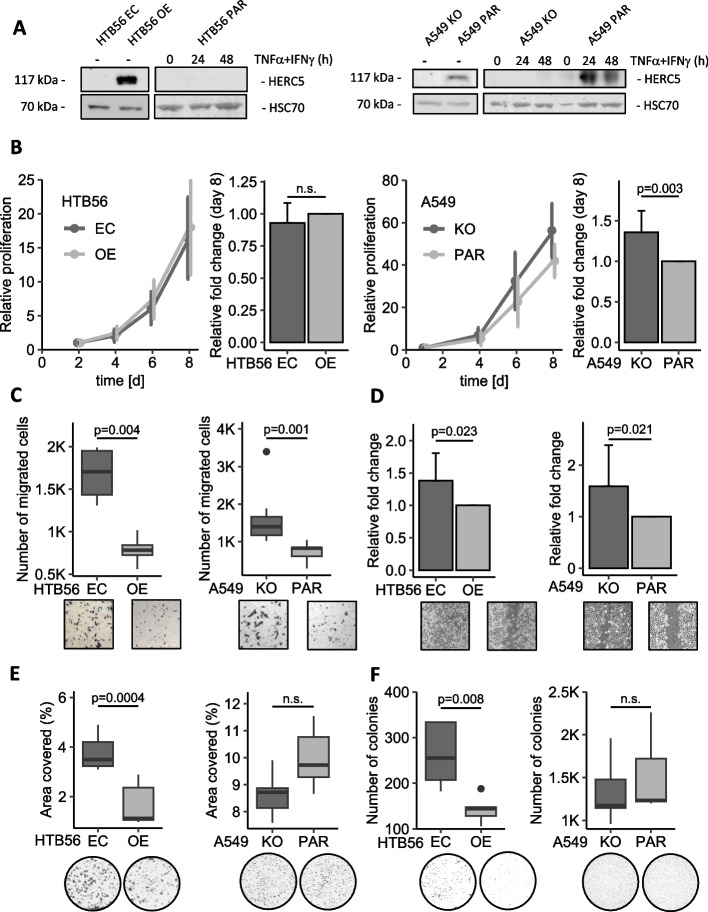
HERC5 inhibits tumor-associated aggressiveness in vitro. **A** Western Blot showing the HERC5 protein levels in the HTB56 OE/EC and A549 PAR/KO model cell lines with and without stimulation. **B** HTB56 EC/OE showed no significant differences in proliferation as assessed by a Methylene Blue proliferation assay (*p* = 0.438, *n* = 6, one sample t-test), while A549 KO/PAR cells showed a significant increase in proliferation at low HERC5 levels (*p* = 0.003, *n* = 11, one sample t-test). **C** HTB56 EC and A549 KO cells have an increased migratory potential compared to HTB56 OE (*p* = 0.004, *n* = 4, Student’s t-test) and A549 PAR cells (*p* = 0.001, *n* = 7, Mann–Whitney U test), respectively. **D** The wound healing assay revealed a higher ratio of migration toward the scratch in HTB56 EC compared to HERC5 OE (*p* = 0.023, *n* = 4, one sample t-test) and A549 HERC5 KO compared to PAR cells (*p* = 0.021, *n* = 3, one sample t-test) after 18 h. **E** and **F** Overexpression of HERC5 caused a decrease in clonogenic and anchorage-independent growth (*p* = 0.0004, *n* = 5, Student’s t-test; *p* = 0.008, *n* = 5, Student’s t-test, respectively) but not in A549 cells (*p* = 0.069, Student’s t-test, *n* = 5; *p* = 0.515, Student’s t-test, *n* = 5, respectively). Error bars in bar plots are depicting the 95% confidence interval

To investigate the impact of HERC5 expression on metastatic traits, we established two different NSCLC cell line models (Fig. 1A) that were selected because of their basal HERC5 expression levels. HTB56 HERC5-overexpressing (OE) and corresponding empty vector control (EC) cell lines as well as A549 parental (PAR) and A549 HERC5 knockout (KO) cell lines were used to identify phenotypical alterations deriving from differential HERC5 expression levels in vitro. The HTB56 parental cell line does not expresses HERC5, whereas the A549 parental cell line does express HERC5.

As HERC5 is best known for its ubiquitin ligase activity mediating ISGylation of protein targets upon activation, we further validated the HERC5 expression status after stimulation with TNFα/IFNγ. As expected, HERC5 expression increases in A549 parental cells, however, did not show upregulation in A549 HERC5 KO and HTB56 cells (Fig. 1A and Fig. S7).

Using Methylene Blue proliferation assays, we detected enhanced proliferation in A549 KO cells compared to parental cells, while no significant difference was detected in the proliferation of the HTB56 cell line between the HERC5 OE and EC cell lines (A549: *p* = 0.003; HTB56: *p* = 0.293; Fig. 1B). The chemotactic behavior of the different cell lines was studied via migration assays using a Transwell chamber assay. The motility of both the HTB56 EC and A549 KO cells was significantly higher than that of the corresponding OE and parental cells (HTB56: *p* = 0.004; A549: *p* = 0.001; Fig. 1C). Furthermore, in wound healing assays, collective cell migration was analyzed, and the results revealed an increase in wound healing in cells with low HERC5 expression (HTB56: *p* = 0.023; A549: *p* = 0.021; Fig. 1D).

Next, the ability of a single clone to grow into a colony in the absence of neighboring cells was analyzed, which revealed an increased potential for colony formation by detached tumor cells in HTB56 EC compared to OE (*p* = 0.0004; Fig. 1E) but not by A549 cells (*p* = 0.069; Fig. 1E). Similarly, soft agar assays revealed enhanced anchorage-independent growth in HTB56 EC compared to OE (*p* = 0.008; Fig. 1F) but not in A549 cells (*p* = 0.515; Fig. 1F).

Overall, the results showed that the absence of the HERC5 protein results in a more aggressive phenotype in NSCLC cells in vitro.

### Low HERC5 expression leads to an increase in dissemination and metastasis formation in vivo

**Fig. 2:**
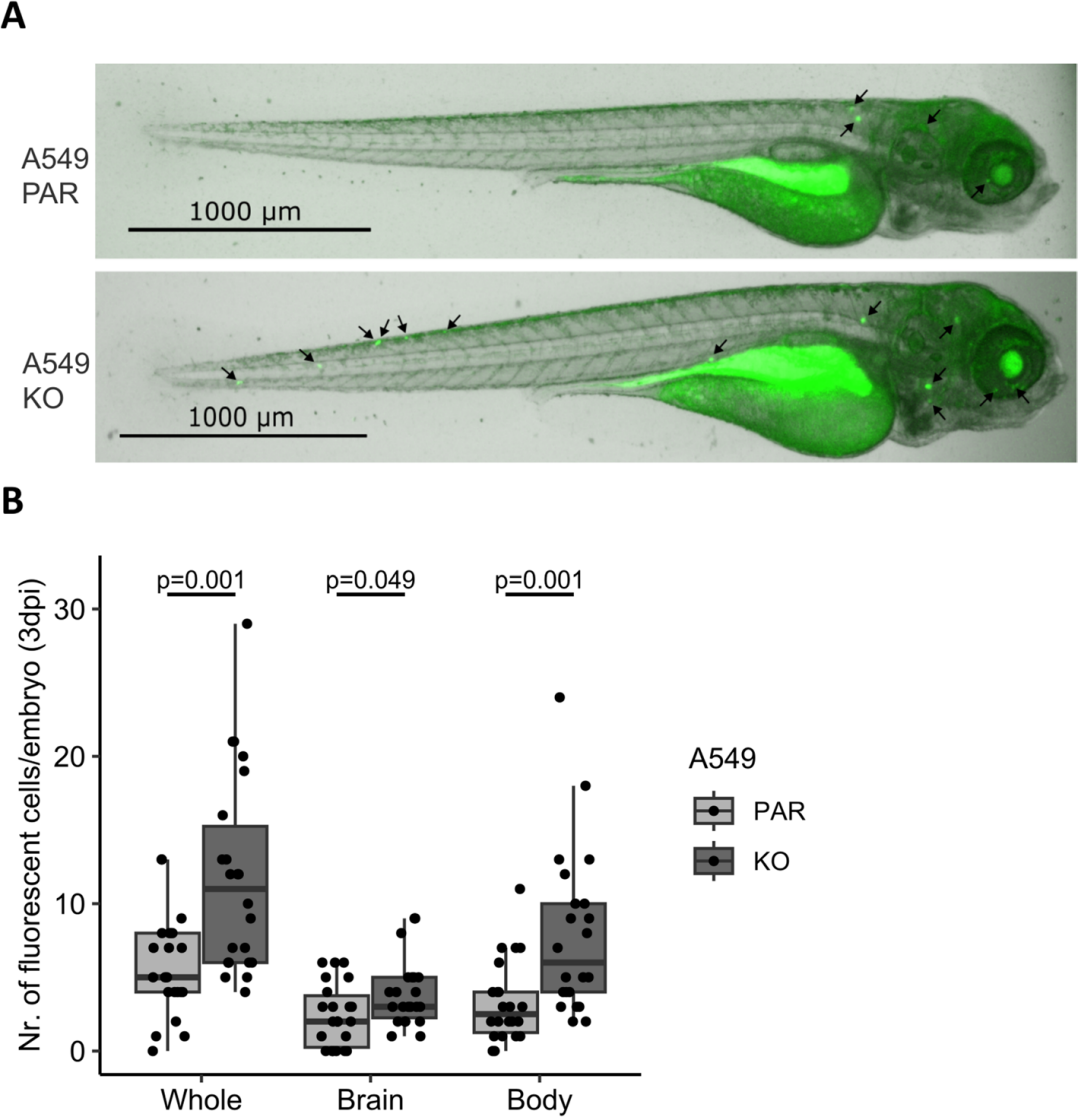
HERC5 KO promotes the survival of DTCs in a zebrafish model. **A** Representative images of zebrafish embryos. The black arrows indicate the fluorescently labeled A549 cells. **B** The number of A549 HERC5 KO cells compared to parental cells per zebrafish embryo is significantly increased in the whole body as well as separately in the brain and the body at 3 days post injection (whole body: *p* = 0.001; brain: *p* = 0.049; body: *p* = 0.001; *n* = 22; Mann–Whitney U Test)

Previously, we reported that loss of HERC5 in NSCLC patients leads to a positive DTC status in the bone marrow of patients with early-stage NSCLC as well as brain metastasis formation [2]. To further investigate the role of HERC5 in tumor cell dissemination and survival in distant tissues, we first carried out in vivo experiments in a zebrafish model. A549 parental and KO cells were injected into the common cardinal vein of fish embryos and monitored at days 1 and 3 postinjection. On day one, there was no significant difference in the total number of cells that had extravasated from the bloodstream between the groups (data not shown). However, on day 3 post injection, the KO group showed a significant increase in the total cell number compared to that of the parental group (*p* = 0.001; Fig. 2B). Given that our clinical data have shown a specific association with brain metastasis formation [2], we also quantified tumor cell numbers in the brains and bodies of zebrafish embryos separately. Figure 2B shows a significantly increased DTC status both in the brain and in the body (brain: *p* = 0.049, body: *p* = 0.001). These results suggest increased survival rates in patients with distant premetastatic sites due to low HERC5 expression and suggest that HERC5 KO contributes to early metastasis formation by enhancing adaptation to the new microenvironment.

Based on these results, we analyzed the effect of HERC5 depletion on tumor cell dissemination and metastasis outgrowth in a mouse model in more detail. For this purpose, we established A549 PAR and KO cells with constitutive luciferase expression (A549 PAR-LUC and A549 HERC5 KO-LUC). These cells were injected intracardially into NMRI-Foxn1^nu^ mice. On day 31, when the first BLI signal above the threshold was identified in the first mouse, all the mice were sacrificed simultaneously.

**Fig. 3:**
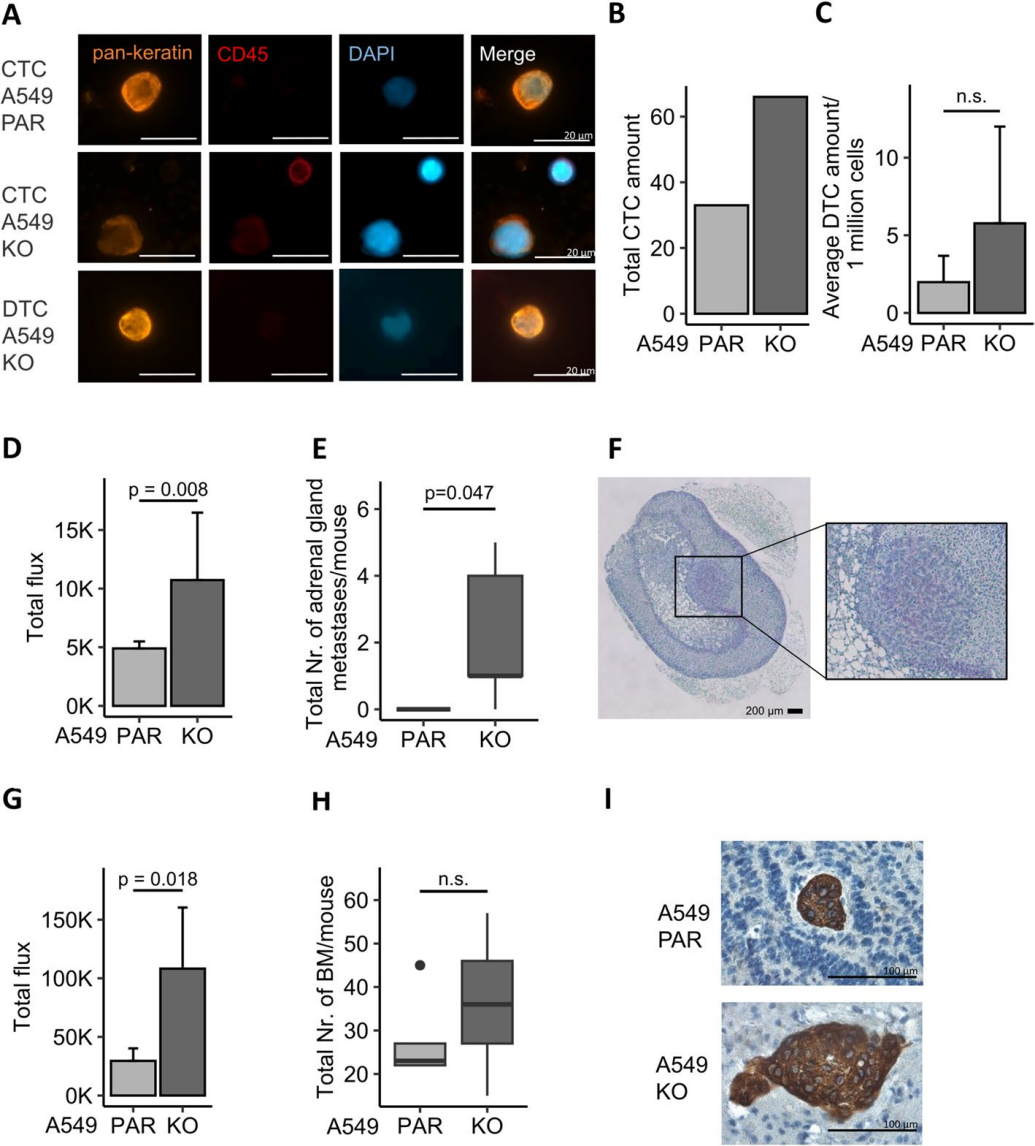
HERC5 inhibits tumor-associated aggressiveness in a mouse model*.* **A** Representative images of CTCs and DTCs detected in murine blood after intracardial injection of 500,000 A549 PAR-LUC and HERC5 KO-LUC cells. Tumor cells were enriched by a size-based enrichment technique and identified by pankeratin positivity, while CD45 was used as an exclusion marker. **B** Total CTC counts in pooled blood from 5 mice from the A549 PAR and HERC5 KO cohorts show an increase in CTCs in those mice injected with A549 HERC5 KO cells. **C** Average DTC amount per 1 million cells is elevated in HERC5 KO cells, however not significantly (*n* = 5). **D** and **E** Adrenal gland metastases were found only in the A549 HERC5 KO cohort; thus, the total number and volume per mouse were significantly increased compared to A549 PAR cohort (*p* = 0.047; *n* = 5; Mann–Whitney U test, respectively). **F** Representative image of adrenal gland metastases identified using HE staining. **G** and **H** The total number and volume of brain metastases per mouse were slightly higher in the A549 KO cohort; however, these differences were not significant (*n* = 5). **I** Representative image of brain metastases identified using pankeratin IHC staining and hematoxylin counterstaining. Error bars in bar plots are depicting the standard deviation

The CTC status was analyzed in pooled blood samples from each cohort. CTCs were identified based on their pattern of epithelial keratin and CD45 expression (Fig. 3A). In total, 66 CTCs were found in the A549 HERC5 KO-LUC group, while 33 CTCs were observed in the A549 PAR-LUC cohort, indicating a two-fold increase in CTCs in those mice that were injected with HERC5 KO cells (Fig. 3B). Furthermore, the analysis of DTCs from the bone marrow of both cohorts revealed an average of 5.77 DTCs/1*10^6^ cells in the HERC5 KO group and 1.98 DTCs/1*10^6^ in the PAR group, with an average fold change increase of 2.91 in the KO mice (*p* = 0.25; Fig. 3C).

Further spreads of metastases to secondary sites and organs were assessed in serial sections of HE- and keratin-stained brain and adrenal gland tissue based on BLI signals. Both the adrenal gland and brain tissue showed a significantly increased BLI signal in the KO group (adrenal gland: *p* = 0.008; brain: *p* = 0.018; Fig. 3D and G). In the PAR cohort, no adrenal gland metastases were identified, while 4 of 5 mice in the HERC5 KO cohort had metastases. Therefore, both the occurrence and volume of adrenal gland metastases were significantly increased in the HERC5 KO cohort than in the PAR cohort (average volume: 22.8 * 10^6^ μm^3^, *p* = 0.047; Fig. 3E, F, and Supplemental Fig. S1A). In brain sections, we detected slight but nonsignificant increases in both the number (41.5 and 27.8, *p* = 0.35) of brain metastases and the total volume (5 * 10^6^ μm^3^ and 2.7 * 10^6^ μm^3^; *p* = 0.54) of metastases in the KO group compared to the PAR group (Fig. 3H, I, and Supplemental Fig. S1B). X-ray analysis of the whole mouse skeleton did not reveal any changes in either group (data not shown).

In vivo, two different models demonstrated that HERC5 deficiency increases the metastatic potential of NSCLC cells, suggesting that low HERC5 expression is associated with improved adaptation to metastatic sites and increased metastatic burden.

### HERC5 induces changes in the mitochondrial protein composition in NSCLC cells

**Fig. 4:**
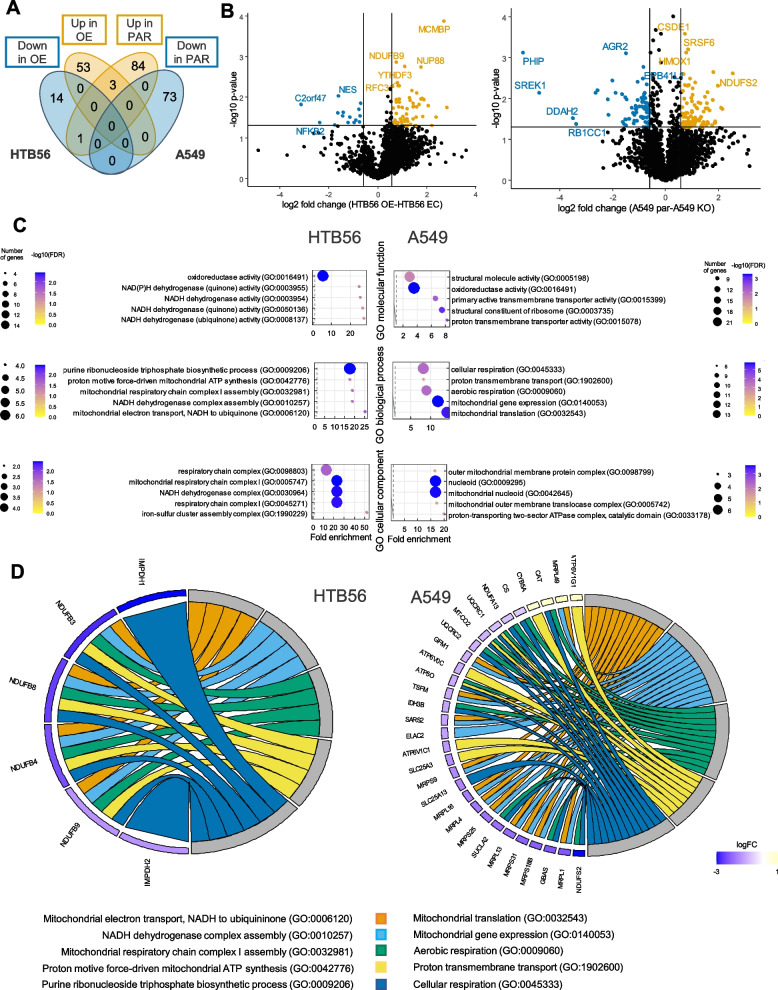
Proteomics reveals enrichment of proteins connected to mitochondrial metabolism in cell lines differentially expressing HERC5. **A** Venn diagram depicting differentially regulated proteins analyzed by mass spectrometry (*n* = 3). Four proteins were found dysregulated in both HTB56 and A549 cell line, with three of them upregulated in cells with high HERC5 expression. **B** Volcano plots depicting differentially expressed proteins (FC > 1.5, *p* value < 0.05, *n* = 3). Significantly downregulated proteins in HTB56 OE and A549 PAR cells are shown in blue, while upregulated proteins are presented in orange. **C** GO term enrichment analysis showing the top five significantly enriched proteins (using R package [37], *n* = 3) **D** Visualization of proteins involved in these GO terms for biological processes by using R package GOplot [38]

To further explore the relationship between loss of HERC5 expression and increased metastatic potential in NSCLC, we investigated changes in the proteome induced by modified HERC5 expression. By comparing A549 PAR with HERC5 KO cells and HTB56 EC with HERC5 OE cells by mass spectrometry, we identified 2445 and 2220 proteins in total with 161 and 71 significantly differentially abundant proteins, respectively (Supplemental Table 1). Of these, 88 proteins were significantly upregulated, while 73 proteins were downregulated in the A549 PAR cells compared to A549 HERC5 KO. Similarly, in the HTB56 HERC5 OE cells, 56 proteins were upregulated, and 15 proteins were downregulated compared to HTB56 EC. Only one (MT-CO2) of these proteins is encoded by mitochondrial DNA. Among the proteins identified in both cell lines, four overlapped (CAV1, MRPL1, CPSF1, and MTHFD2), and 3 of these differentially expressed proteins correlated with high HERC5 expression (CAV1, MRPL1, and CPSF1; Fig. 4A and B). Despite the low number of overlapping proteins in the cell lines, GO term analyses of the top five fold-change enriched proteins showed a striking similarity to the most commonly and significantly enriched terms in the molecular function, biological process, and cellular component categories (Fig. 4C, Supplemental Fig. S2A and B, Supplemental Table 2). Overall, significant enrichment was detected in terms of mitochondrial metabolism and NADH dehydrogenase complex activity (Fig. 4C). Furthermore, the relationships between the GO biological processes and the corresponding genes involved were visualized. In HTB56 cells, the main group of genes involved in the top five enriched biological processes was NADH dehydrogenases, while in A549 cells, mitochondrial ribosomal proteins were also observed (Fig. 4D).

To analyze whether the observed association with mitochondrial metabolism is independent from its function as a ubiquitin ligase, we performed the MS experiments with stimulated A549 parental and KO cell line models. For this, we analyzed the expression of HERC5, ISG15 and ISGylated proteins on western blots upon TNFα/IFNγ stimulation, showing a clear induction of both HERC5 and ISGylated proteins in the A549 PAR, but not in the KO cells. No upregulation of either HERC5 or ISG15 levels in HTB56 cells could be observed (Supplemental Figs. S7 and S8). The MS analysis identified 102 differently expressed proteins between the parental and KO stimulated A549 cell lines. Of these, 41 proteins were upregulated and 61 proteins were downregulated in the A549 par cells (Supplemental Table 1). GO term analysis showed mainly enrichment in nuclear proteins and did not reveal any significant enrichment in any terms connected to mitochondrial metabolism (Supplemental Fig. S3A and Supplemental Table 3). When comparing the untreated with treated cells, major differences in interferon and interleukin signaling, antigen presentation and antiviral defense were observed (Supplemental Fig. S3B and Supplemental Table 3).

To furthermore analyze whether the obtained results are only cell line specific, we performed an *in silico* validation analysis using a publicly available NSCLC MS data set [36]. Here we identified 241 and 143 proteins that were significantly differentially regulated (≥ 1.5 FC) in patients with either low or high HERC5 expression, respectively. Identification of associated GO terms by enrichment analysis revealed a significant overrepresentation of proteins connected to viral responses and innate immunity in patients with high HERC5 expression. In contrast, in patients with low HERC5 expression significant proteins were associated with cell cycle, migration, metabolism, NADH dehydrogenase activity, and mitochondrial function, recapitulating the cell line results for untreated cells (Supplemental Fig. S4). In patients with high HERC5 expression, ISG15 and USP18 (ISG15 de-ubiquitinylase) were among the top 10 regulated proteins, which are the main interaction partners of HERC5 mediated ISGylation. In patients with low expression on the other hand, the most strongly regulated protein was SLC16A4, which has been recently described to be involved in alterations of glucose and lactate metabolism and metastasis also in NSCLC [41–43]. Overall, cell line and patient data indicate that in NSCLC the HERC5-associated alterations in mitochondrial metabolism are independent of its ISGylation function.

### HERC5 shifts ATP production from glycolysis to oxidative phosphorylation

**Fig. 5:**
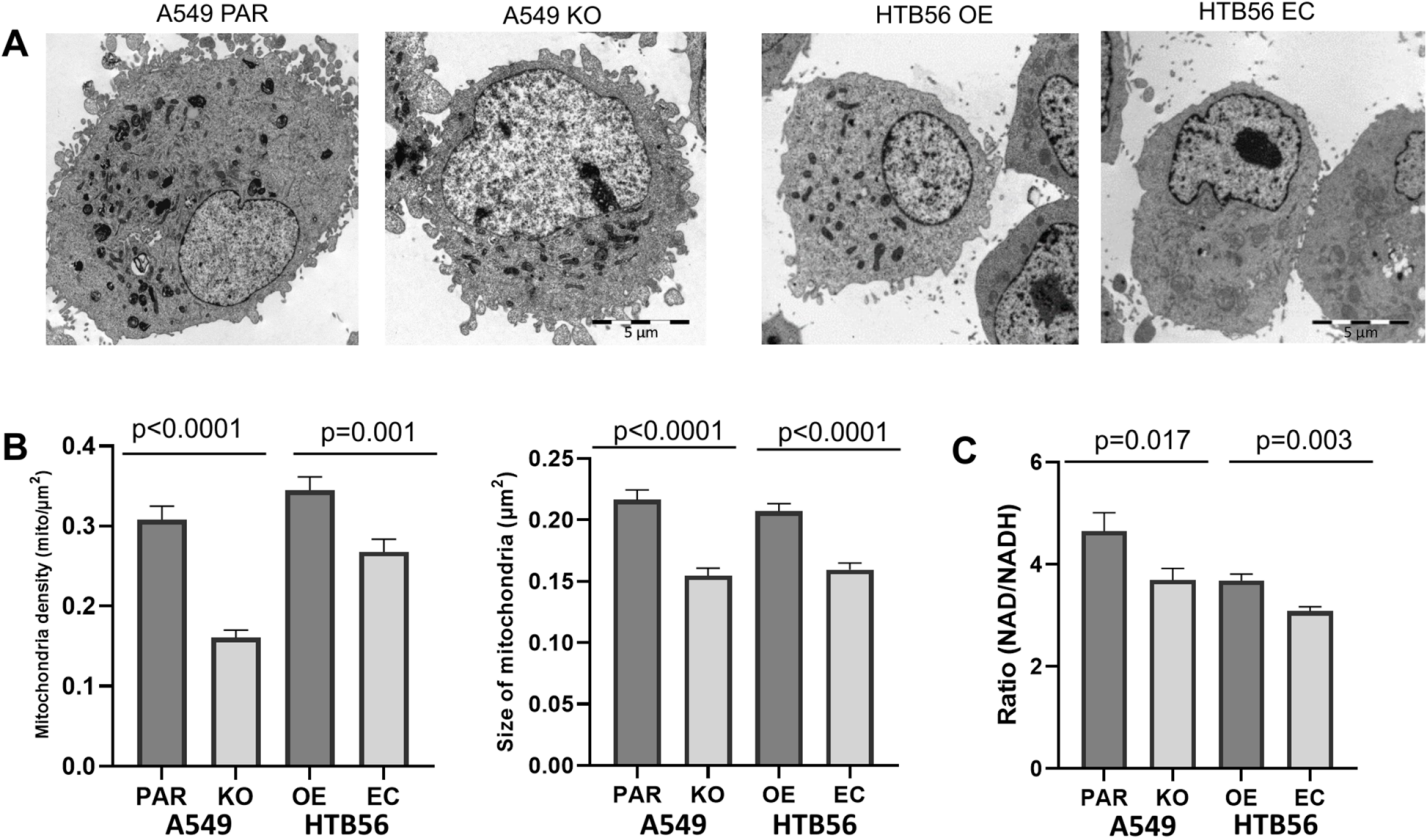
HERC5 induces changes in mitochondrial morphology. **A** Representative electron microscopy images of A549 PAR/KO and HTB56 OE/EC cells. **B** Mitochondrial density and size increase in cells with high HERC5 expression (density: HTB56 EC/OE: *p* = 0.001; A549 KO/PAR: *p* < 0.0001; *n* = 30; size: HTB56 EC/OE: *p* < 0.0001, *n* = 490/578; A549 KO/PAR: *p* < 0.0001, *n* = 337/395; Student’s T-test). **C** HTB56 EC and A549 HERC5 KO have a decreased NAD/NADH ratio compared to HTB56 HERC5 OE (*p* = 0.003, *n* = 3, Student’s T-test) and A549 PAR (*p* = 0.017, *n* = 3, Student’s T-test) cells, respectively. Error bars in bar plots are depicting the standard deviation

Due to the high number of differentially regulated proteins related to mitochondrial function in NSCLC cell lines, we analyzed whether there were numerical and morphological changes in the mitochondria of these cell lines. Using electron microscopy, significant increases in mitochondrial density and size were observed in cells with high HERC5 expression (A549 PAR versus KO: density: *p* < 0.0001, size: *p* < 0.0001; HTB56 OE versus EC: density: *p* = 0.001, size: *p* < 0.0001; Fig. 5A and B). As migration and invasion are highly energy-consuming processes, we stained our cells in the wound healing assay with MitoTrackers. The cells in the migrating front showed high activation of mitochondrial activity with a directed lamellipodia-like movement pattern. However, no differences between the cell lines were observed (Supplemental Fig. S5).

To further investigate the consequences of modified HERC5 expression on phenotypical differences in mitochondrial protein composition and function, we analyzed the ratio of NAD to NADH in the cells, which might correlate with the activity of NADH dehydrogenases. HTB56 EC and A549 HERC5 KO cells had decreased NAD/NADH ratios compared to HTB56 HERC5 OE (*p* = 0.003) and A549 PAR (*p* = 0.017) cells, correlating with the overexpression of numerous NADH dehydrogenases in cell lines with high HERC5 expression (Figs. 4C, D, and 5C).

**Fig. 6:**
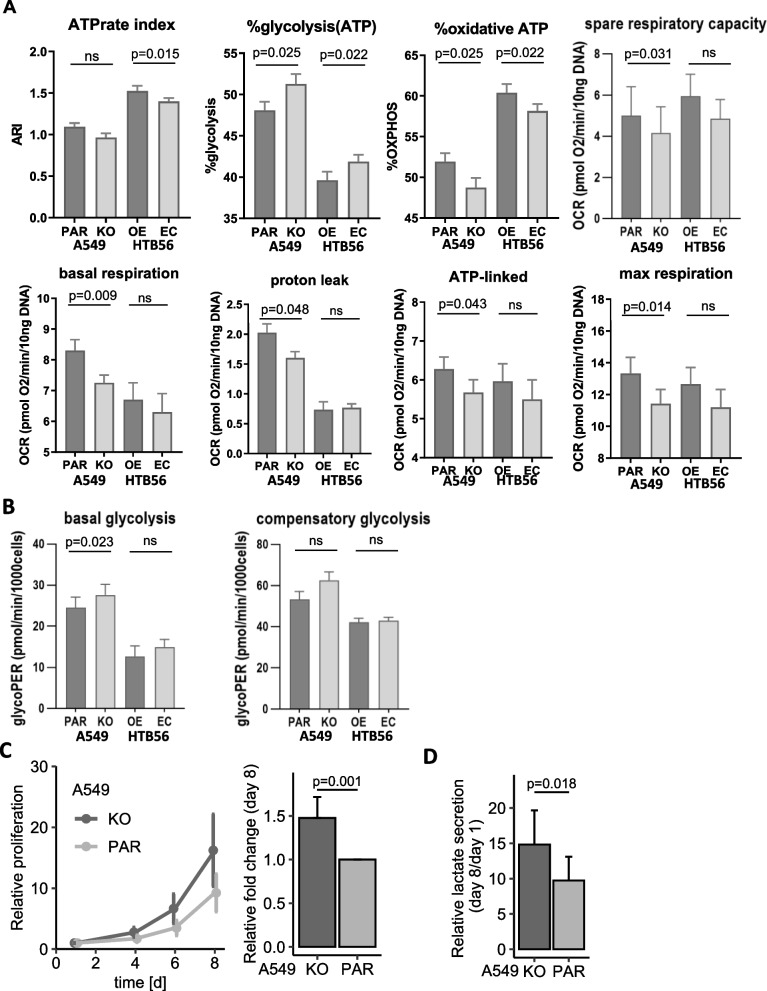
HERC5 KO switches metabolism from oxidative phosphorylation to aerobic glycolysis. **A** Loss of HERC5 expression shifts the ratio of ATP production in the cell to a decrease in mitochondrial energy production and toward glycolysis. The ATP rate index, as well as the ratio of glycolytic and oxidative ATP production, was altered in A549 PAR and HTB56 OE cells (ATPrate index: A549 KO/PAR: *p* = 0.082; HTB56 EC/OE: *p* = 0.015; glycolytic ATP: A549 KO/PAR: *p* = 0.025; HTB56 EC/OE: *p* = 0.022; oxidative ATP: A549 KO/PAR: *p* = 0.025; HTB56 EC/OE: *p* = 0.022; Paired t-test). Seahorse Mito stress assays also revealed an increase in basal respiration (*p* = 0.009; *p* = 0.314; Paired t-test), proton leak (*p* = 0.048; *p* = 0.667; Paired t-test), ATP-linked respiration (*p* = 0.043; *p* = 0.331; Paired t-test) and maximal respiration (*p* = 0.014; *p* = 0.274; Paired t-test) in A549 PAR cells compared to HERC5 KO cells and HTB56 HERC5 OE compared to EC cells, respectively. All the experiments were performed with *n* = 4. Error bars depict the standard deviation. **B** Glycolytic Rate Assay shows significant increase in basal glycolysis (*p* = 0.023, Paired t-test) and increase, although not significantly (*p* = 0.053, Paired t-test), compensatory glycolysis of A549 HERC5 KO cells compared to A549 PAR. There was no significant effect in the HTB56 HERC5 OE cell model. The Assay was performed with *n* = 4. Upon long-term treatment (8 days) with the ATP synthase inhibitor oligomycin (100 ng/mL), A549 KO/PAR cells showed **C** significantly elevated proliferation (*p* = 0.001, *n* = 11, one sample t-test) and **D** significantly elevated lactate levels in the supernatant of HERC5 KO cells (*p* = 0.018, *n* = 8, Paired t-test). Error bars are depicting the 95% confidence interval

To analyze whether the observed quantitative differences in mitochondrial morphology and protein levels also result in changes in energy metabolism, we determined cellular respiration and glycolysis using a Seahorse XF analyzer. Here, we showed that low HERC5 expression correlated with an increase in ATP production through glycolysis, whereas high HERC5 expression led to increased ATP generation through oxidative pathways, indicating a link between HERC5 and the Warburg effect (Fig. 6A; A549 KO/PAR: *p* = 0.025; HTB56 EC/OE: *p* = 0.022; *n* = 4). Moreover, in line with the increased mitochondrial density and size in A549 parental cells compared to A549 HERC5 KO cells, the overall respiration capacity was increased, as shown by the basal respiration, proton leak, ATP-linked respiration, maximal respiration, and spare respiratory capacity (Fig. 6A; *p* = 0.009, *p* = 0.048, *p* = 0.043, *p* = 0.014, and *p* = 0.031, respectively; *n* = 4). The Seahorse XF Glycolytic Rate analysis showed a significant increase in basal glycolysis and borderline significant increase in compensatory glycolysis in the A549 HERC5 KO cell line compared to A549 PAR (Fig. 6B; basal glycolysis: *p* = 0.023; compensatory glycolysis: *p* = 0.053). The HTB56 HERC5 OE cell line model showed borderline significant increase in basal glycolysis but no effect in compensatory glycolysis (Fig. 6B; basal glycolysis: *p* = 0.056; compensatory glycolysis: *p* = 0.499). Altogether, these results support our MS analysis of a change in glucose metabolism due to low HERC5 expression. Based on the enhanced survival rates and increased early metastasis formation in the zebrafish model, we furthermore analyzed whether the aggressive phenotype observed in A549 HERC5 KO-LUC cells results from an adaptation to stress conditions. We therefore measured in vitro cell proliferation and lactate secretion under long-term hypoxic or OXPHOS-suppressing conditions. Here, we report that proliferation is not altered in a hypoxic environment (Supplemental Fig. S4B), while proliferation and lactate secretion are increased in A549 HERC5 KO cells compared to parental cells using the OXPHOS inhibitor oligomycin (proliferation: *p* = 0.001; lactate secretion: *p* = 0.018; Fig. 6C and D). In summary, loss of HERC5 leads to a metabolic switch, which is one of the hallmarks of cancer; this switch favors the suppression of normal oxidative phosphorylation (OXPHOS) and the adaptation to hypoxia.

## Discussion

Early metastatic spread of lung cancer remains the main reason for its high mortality rate. To understand the critical steps necessary for successful metastasis formation, more in-depth knowledge of the role of metastasis suppressor genes, epigenetics, and crosstalk with the tumor microenvironment is needed. Previously, we and others have identified tumor and metastasis suppressive functions of HERC5 in different tumor entities [2–4]. In NSCLC patients, HERC5 was shown to be involved in early dissemination and brain metastasis formation [2]. While HERC5 has thus far been described mainly in the context of ISGylation and innate immune responses [11, 12], here, we show, for the first time, that HERC5 also directly promotes carcinogenicity and metastasis in NSCLC mainly through a switch in energy metabolism, which is independent of its ISGylation function.

Using functional in vitro assays, we showed that knocking out HERC5 leads to an increase in migratory potential, colony formation, anchorage-independent growth, and invasive ability, thereby demonstrating its metastasis-suppressing function.

We further analyzed the role of HERC5 in extravasation, survival in distant tissues, and metastasis formation in vivo. Therefore, we used both a zebrafish and a mouse model. We used CellTracker-stained HERC5 KO and parental cell lines to visualize metastasis dynamics at early stages in zebrafish. We showed that extravasation is not affected by HERC5 expression. However, on day 3 postinjection, more HERC5 KO cells were detected both in the body and in the brain, thus showing that loss of HERC5 promotes the survival of disseminated cells in vivo in distant organs such as the brain. As all models, the zebrafish embryo xenograft model has some limitations. It is a simplified cancer model with species difference, immature organ systems and short-duration of experiments [44]. Despite these limitations, it has been successfully used to study several aspects of cancer biology [44, 45], but some caution is needed in interpretation of the results. To further analyze the metastatic potential of HERC5 in vivo, we used a model of intracardial injection of an NSCLC parental and HERC5 KO cell line in immune-deficient mice. The HERC5 gene consists of conserved sequences across various species that have not changed much throughout evolutionary history [46–48]. However, the main known function of HERC5 as the E3 ligase for ISG15 is not conserved in mice; instead, this function is carried out by murine HERC6 [49]. Therefore, using HERC5 KO mice would not have reflected the common traits between these two gene analogs. Here, we showed that the dissemination of tumor cells is increased in HERC5 KO cells in comparison to that in their parental counterpart, causing an increase mainly in the adrenal gland and brain metastases. Thus, HERC5 downregulation is responsible for metastasis formation in vivo*,* leading to earlier and more aggressive tumor dissemination. These findings are in line with our previous studies showing that loss of the HERC5 locus is associated with bone marrow-positive DTC status, brain metastasis, and poor prognosis in patients [50]. Furthermore, as both in vitro and in vivo experiments were performed without activation of innate immune responses, this phenomenon seems to be independent of its potential ISG15-related immune defense mechanism.

To validate how HERC5 confers metastatic traits within cells, we analyzed the whole proteome of NSCLC cell lines with differential HERC5 expression by mass spectrometry and found a remarkable overlap of proteins that have altered expression of mitochondrial energy metabolism pathways in both cell lines. Furthermore, by performing MS analyses of cell lines in which ISGylation was induced, we could show that the GO terms of the enriched proteins are not related to mitochondrial metabolism. These features can also be observed in in silico analyses from NSCLC patient data. Therefore, we conclude that alterations in energy metabolism induced by HERC5 is independent from its involvement in innate immune responses.

Interestingly, both mitochondrial density and size are diminished in cells that do not express HERC5. Within the cell, mitochondria are organelles that build networks similar to those of the endoplasmic reticulum and are constantly undergoing fission and fusion processes to rapidly meet the demands of cells under various energetic and metabolic conditions [51]. During migration, mitochondrial fission occurs more predominantly, and mitochondria are transported along filaments to the migratory front to provide ATP for this highly energetic process [52, 53]. Thus, smaller, fractured mitochondria are related to increased migratory behavior and thus aid in metastatic spread, in which high motility is beneficial for cancer cell survival [53–56]. Both traits can be observed in our HERC5 KO cells.

Furthermore, a high number of differentially regulated NADH dehydrogenases, such as NDUFS2, were identified. These crucial enzymes in complex I of the OXPHOS pathway are responsible for reducing NADH to NAD to generate electrons, which are ultimately metabolized for ATP generation [57]. Here, cells with no HERC5 expression indeed had a decreased NAD/NADH ratio. This imbalance could lead to HERC5-dependent impairment of complex I of the electron transport chain, decreasing the efficiency of the OXPHOS pathway and decreasing ATP production in the mitochondria. NADH dehydrogenases have previously been implicated in cancer and metastasis formation due to their central role in the NAD/NADH redox balance and OXPHOS. Their activity was found to decrease, e.g., in breast cancer and CRC cells, which exhibit the Warburg effect, leading to the inhibition of mitochondrial activity and thereby promoting metastatic growth [58–60].

In addition, to the expression of NADH dehydrogenases, the expression of HERC5 is correlated with the expression of several other key players involved in OXPHOS genes and proteins located in mitochondria. To further support our findings, we subsequently analyzed changes in cellular energy metabolism dependent on HERC5 expression. Therefore, we determined the maximal capacity of the cells to execute respiration or glycolysis under stress conditions. Furthermore, we determined the ATP production through either glycolysis or oxidative phosphorylation under basal conditions. Here, we showed for the first time that cells with low HERC5 expression undergo a shift toward glycolytic ATP production rather than cells with high levels of HERC5, which results in the generation of more ATP through oxidative pathways. Additionally, we showed that HERC5-expressing cells exhibit an overall increase in oxidative respiration. In reverse we observed an increase in the basal glycolysis in low-HERC5-expressing cells using the Seahorse XF Analyzer. Although, compensatory glycolysis was not significantly increased in our cell line models we observed a trend towards increased compensatory glycolysis in the A549 HERC5 KO cells. Together, with a higher proliferation rate and lactate secretion over long time oligomycin-induced OXPHOS inhibition, these findings reveal that HERC5 KO cells are changing their metabolism towards glycolysis and are more effective at adapting to severe energy stress. Moreover, we observed no effect of long-term hypoxia-induced OXPHOS inhibition. In contrast to oligomycin, hypoxia induces a metabolic switch through several mechanisms by increasing glycolysis through, e.g., the HIF pathway and posttranslational modifications of glycolytic enzymes, which in sum could compensate for the effect contributed by the loss of HERC5 [28, 61, 62]. Taken together, our results suggest a link between HERC5 and the Warburg effect. The Warburg effect is a generally well-described important cancer-associated trait that contributes to several mechanisms of tumorigenesis and metastasis formation [63].

## Conclusions

In summary, we provide new evidence that HERC5 acts as a metastasis suppressor gene in NSCLC by altering the metabolic state of cells. Its loss causes cells to switch from an oxidative to a more glycolytic state, indicative of the Warburg effect, thereby promoting cell migration and metastatic growth.

### Supplementary Information

**Additional file 1.** Supplemental Figures, Images and graphs showing supporting results.

**Additional file 2:** **Supplemental Table 1.** Mass Spectrometry data of differentially expressed proteins. List containing all differentially expressed proteins detected by Mass Spectrometry (*p*< 0.05) in A549 parental vs. HERC5 KO and HTB56 EC vs. HERC5 OE, A549 PAR treated vs KO treated, A549 untreated vs. treated and cell lines.

**Additional file 3:** **Supplemental Table 2.** Panther Overrepresentation Test of differentially expressed proteins. List containing all GO terms enriched in A549 parental vs. HERC5 KO and HTB56 EC vs. HERC5 OE cell lines.

**Supplementary file 4:** **Supplemental Table 3.** Panther Overrepresentation Test of differentially expressed proteins. List containing all GO terms enriched in A549 PAR treated vs KO treated, and A549 untreated vs. treated cell lines.

## Data Availability

MS data are available via ProteomeXchange with the identifier PXD045563. Further datasets generated and analyzed during the current study are available from the corresponding author upon reasonable request.

## Abbreviations

BLI: Bioluminescent Imaging
CTC: Circulating Tumor Cell
Dpi: Days postinjection
DTC: Disseminated Tumor Cell
EC: Empty vector control
HERC5: HECT and RLD Domain Containing E3 Ubiquitin Protein Ligase 5
ISG15: Interferon-Stimulated Gene 15
KO: Knockout
NSCLC: Non-Small Cell Lung Cancer
OE: Overexpression
OS: Overall survival
OXPHOS: Oxidative phosphorylation
PAR: Parental
