# Supplementary material for: HERC5 downregulation in non-small cell lung cancer is associated with altered energy metabolism and metastasis
Source: J Exp Clin Cancer Res. 2024 Apr 11;43:110. doi: 10.1186/s13046-024-03020-z (PMC11008035; doi:10.1186/s13046-024-03020-z)
Supplement: Supplementary file 1 — Additional file 1. Supplemental Figures, Images and graphs showing supporting results. [file 13046_2024_3020_MOESM1_ESM.docx]

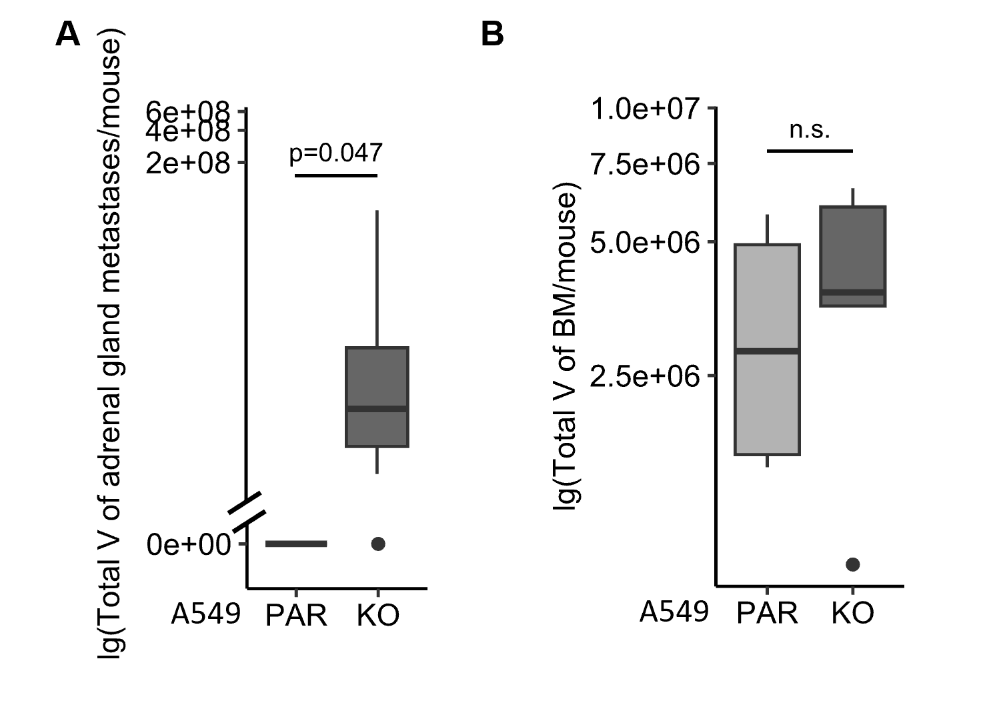


Supplemental Figure S1


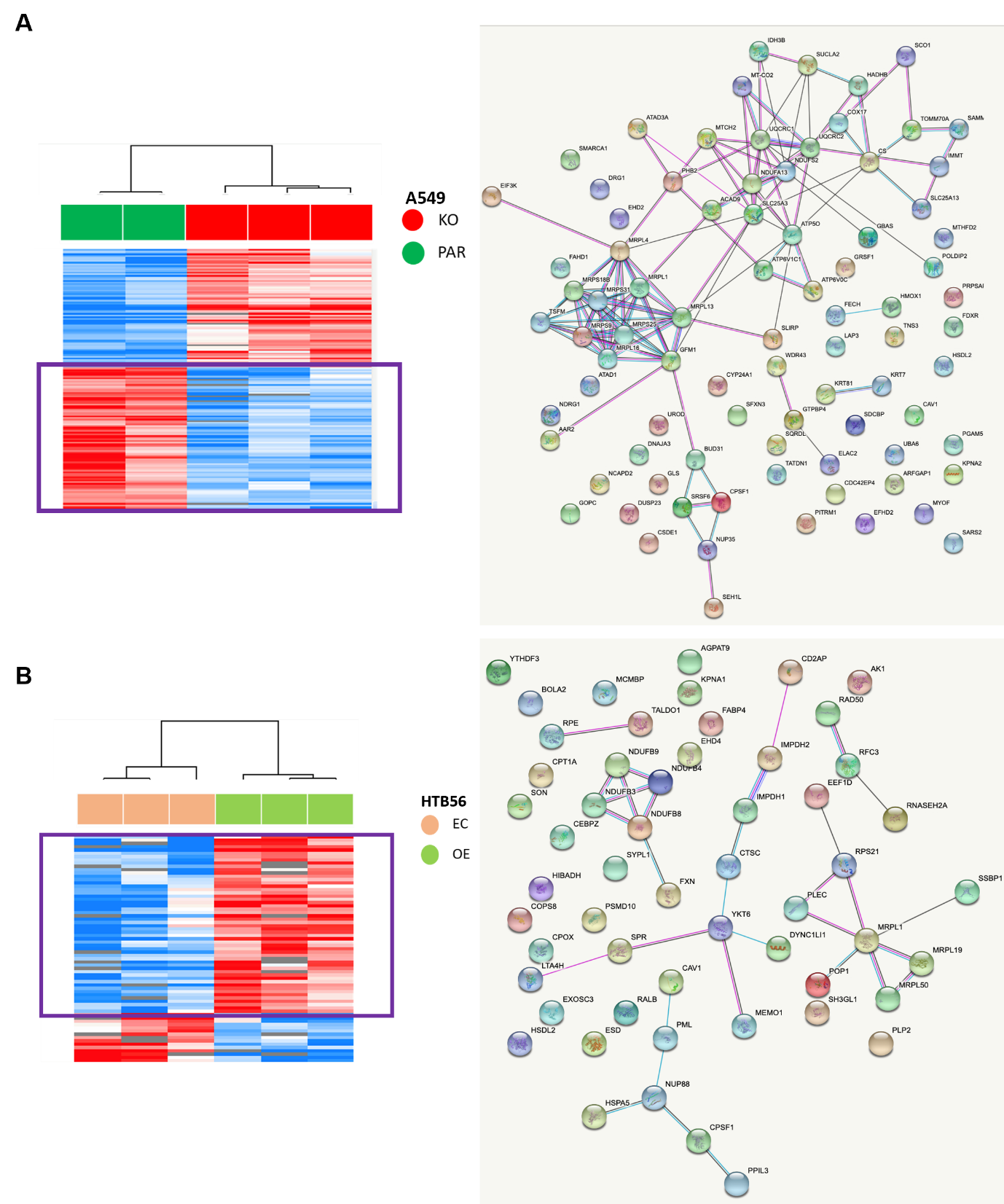


Supplemental Figure S2


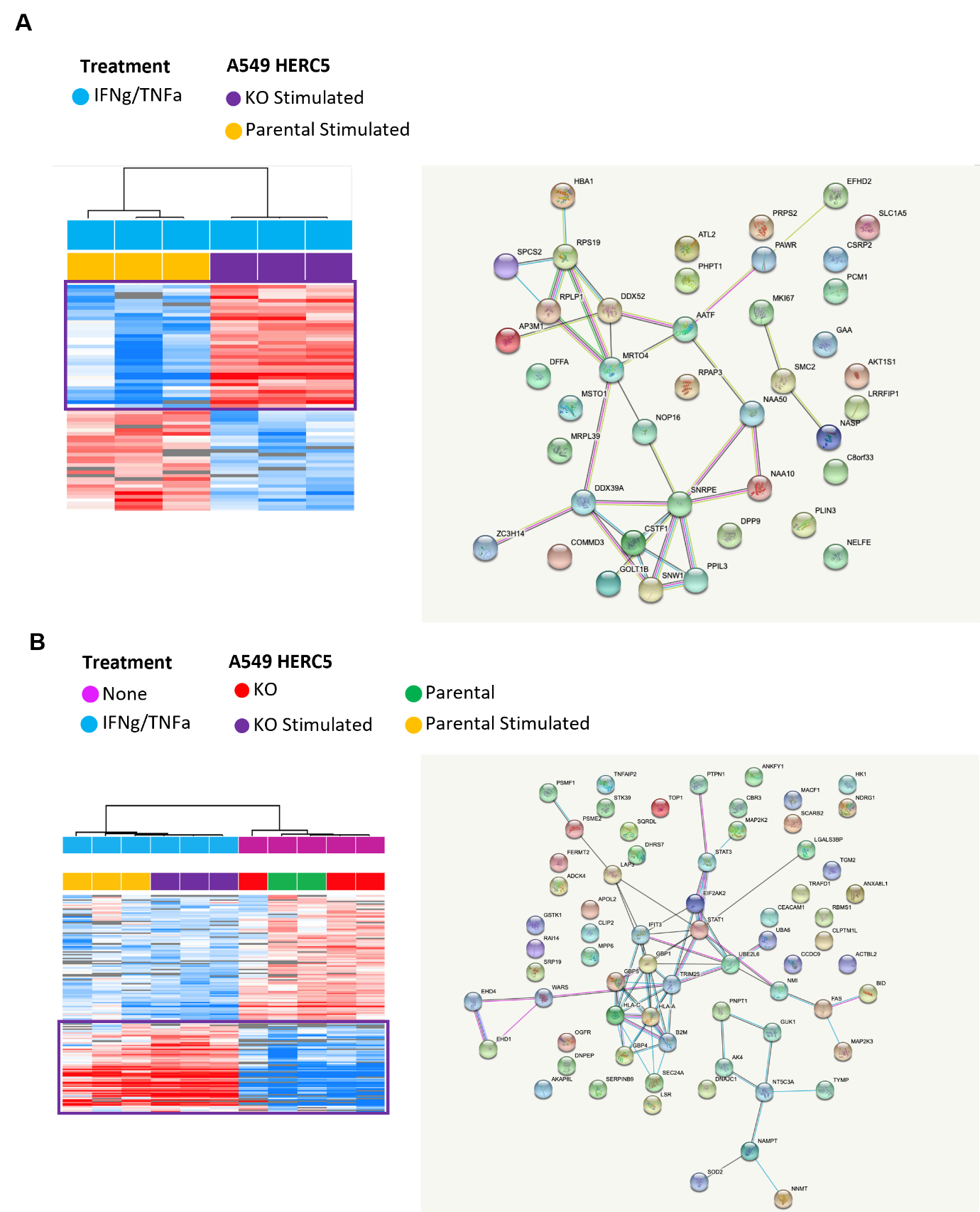


Supplemental Figure S3


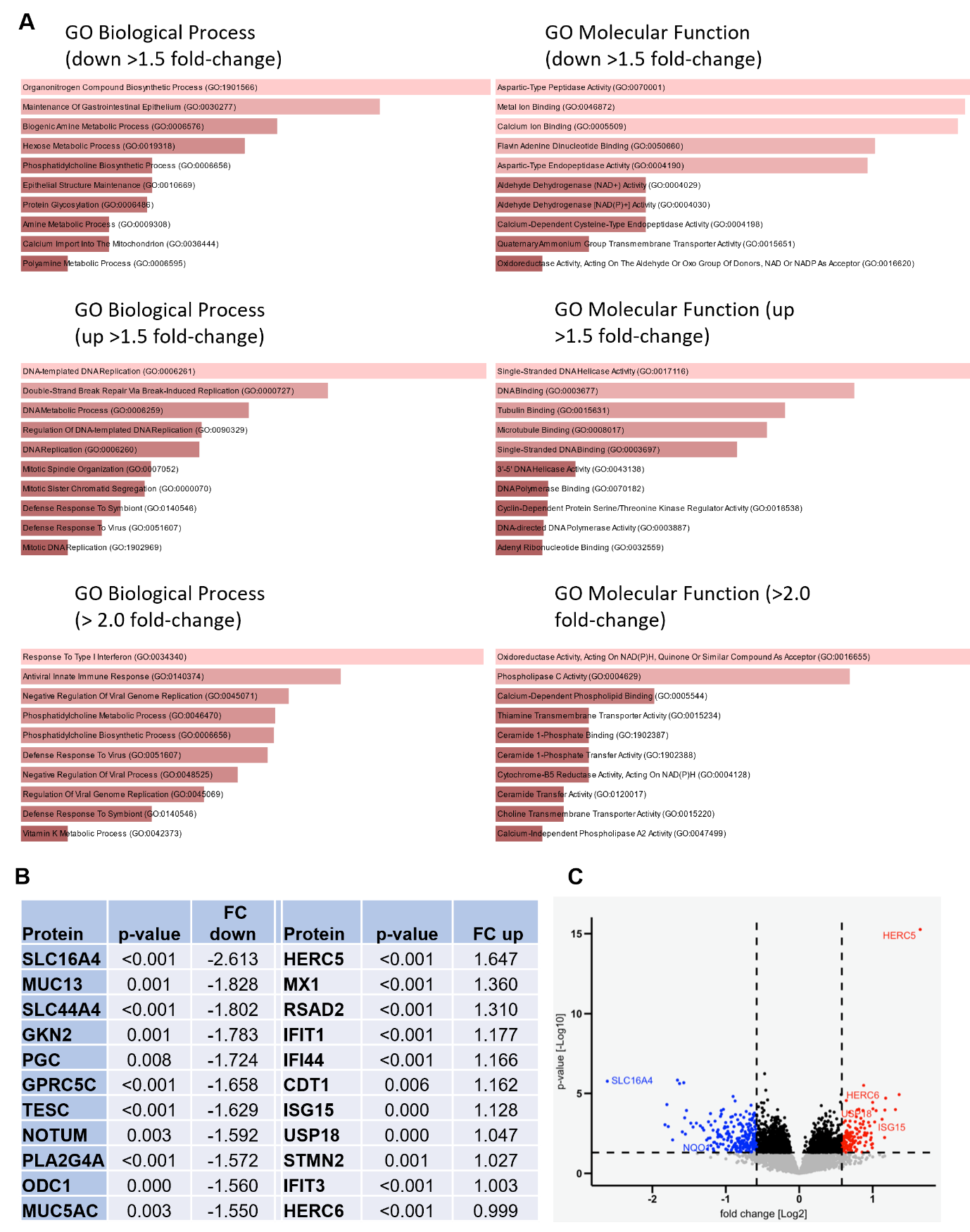


Supplemental Figure S4


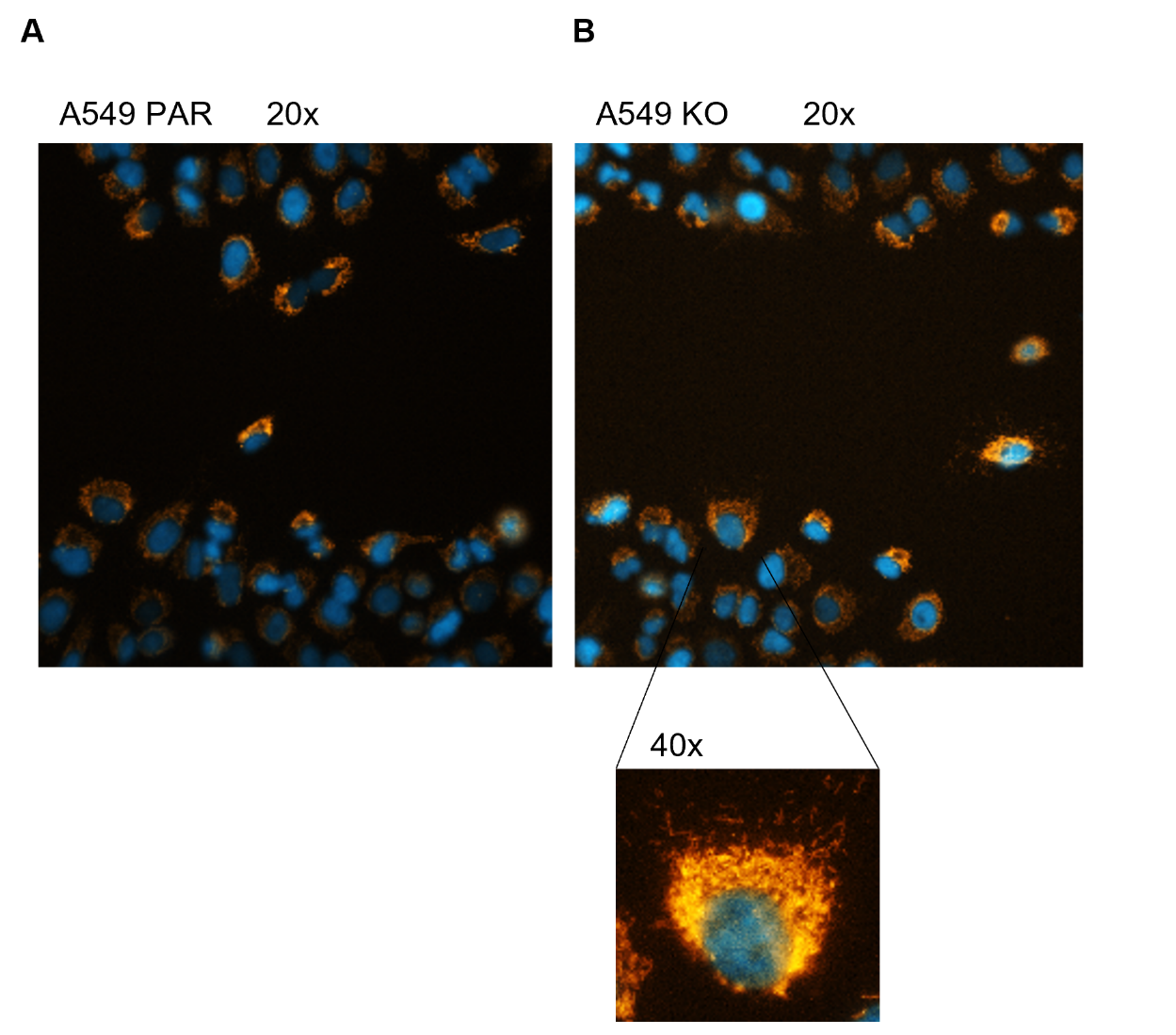


Supplemental Figure S5


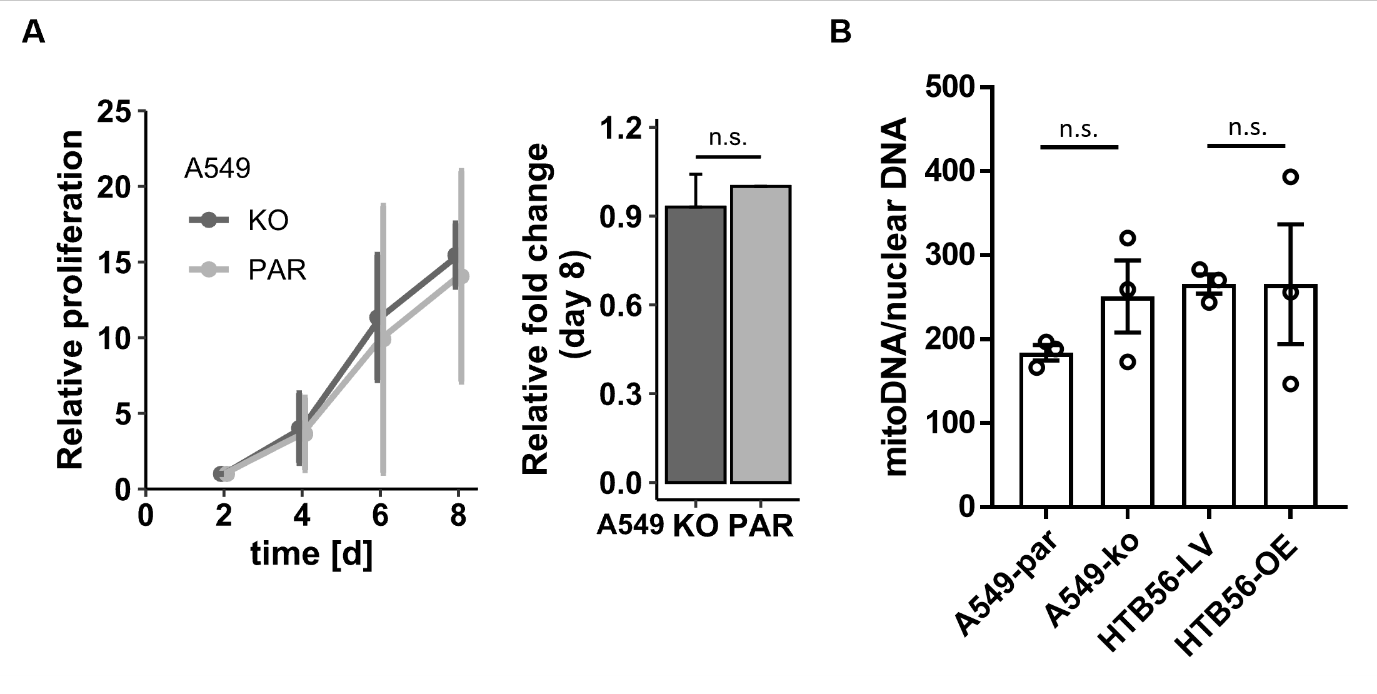


Supplemental Figure S6


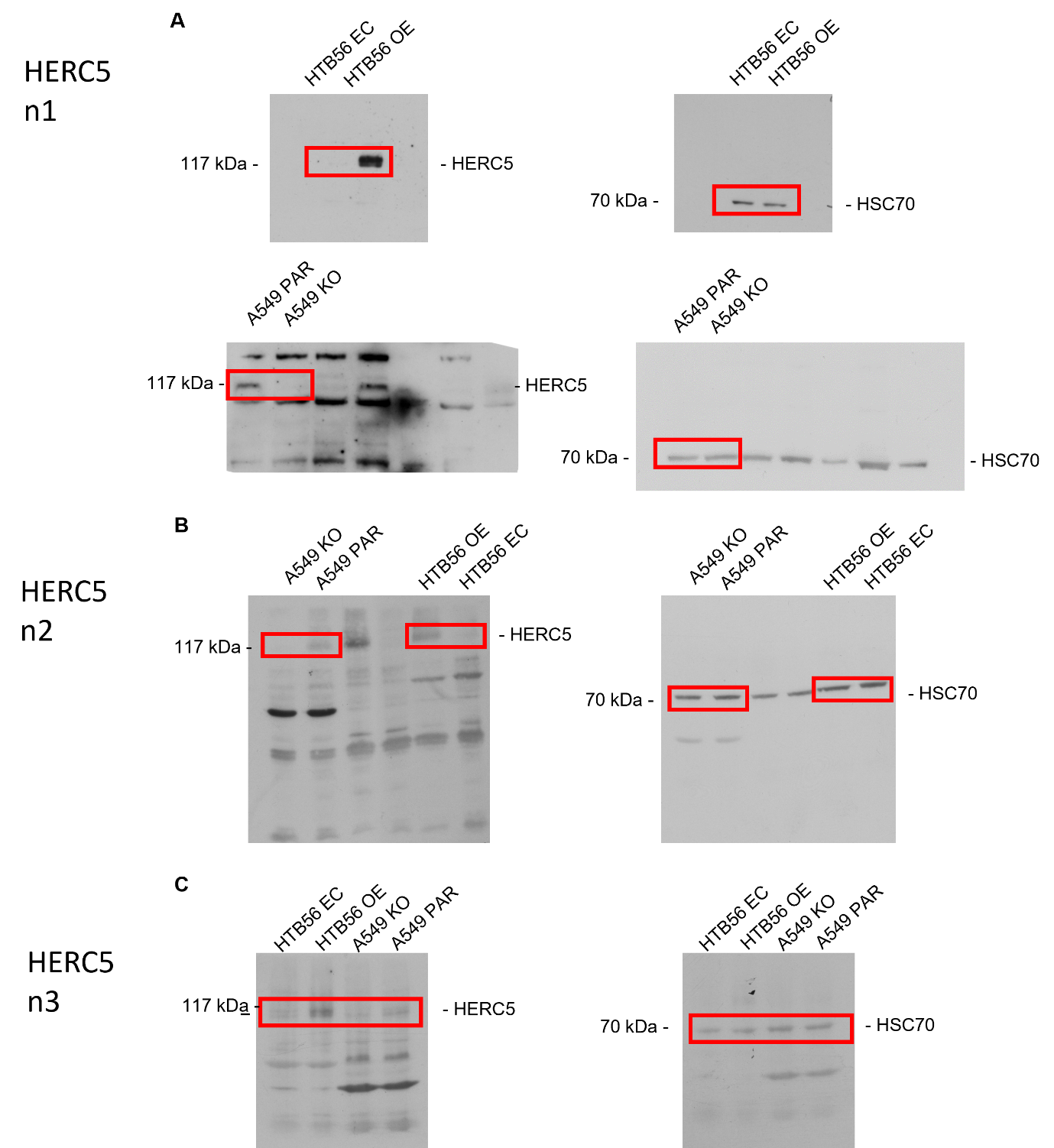


Supplemental Figure S7


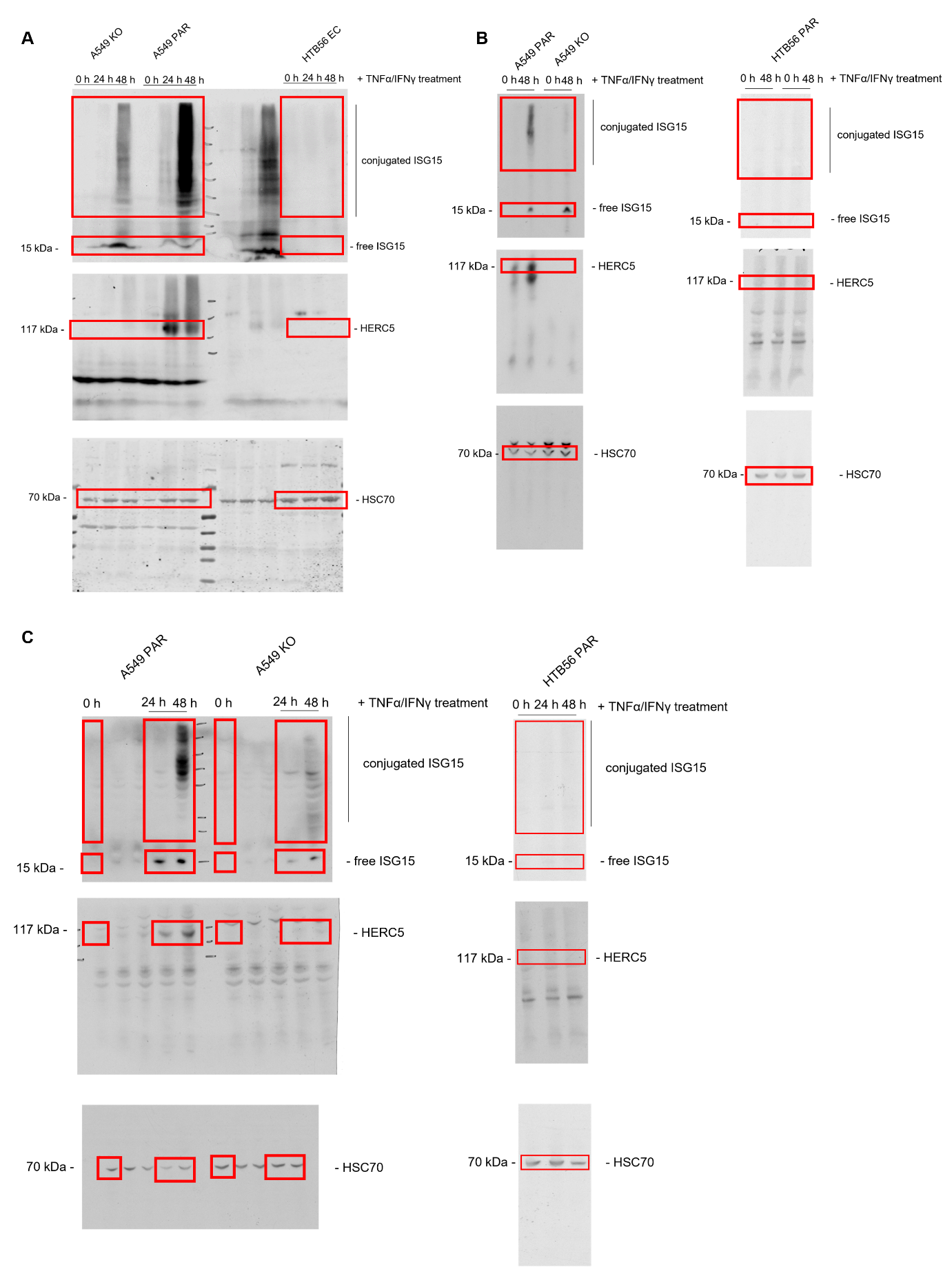


Supplemental Figure S8
